# The association between neutrophil counts and neutrophil-to-lymphocyte ratio and stress hyperglycemia in patients with acute ischemic stroke according to stroke etiology

**DOI:** 10.3389/fendo.2023.1117408

**Published:** 2023-03-16

**Authors:** Xianjing Feng, Fang Yu, Minping Wei, Yunfang Luo, Tingting Zhao, Zeyu Liu, Qin Huang, Ruxin Tu, Jiaxin Li, Boxin Zhang, Liuyang Cheng, Jian Xia

**Affiliations:** ^1^ Department of Neurology, Xiangya Hospital, Central South University, Changsha, Hunan, China; ^2^ Clinical Research Center for Cerebrovascular Disease of Hunan Province, Central South University, Changsha, Hunan, China; ^3^ National Clinical Research Center for Geriatric Disorders, Xiangya Hospital, Central South University, Changsha, Hunan, China

**Keywords:** ischemic stroke, stress hyperglycemia, neutrophil, lymphocyte-to-monocyte ratio, stroke etiology

## Abstract

**Background and purpose:**

Stress hyperglycemia ratio (SHR), which is used to assess stress hyperglycemia, is associated with the functional outcome of ischemic stroke (IS). IS can induce the inflammatory response. Neutrophil counts and neutrophil-to-lymphocyte ratio (NLR) as good and easily available inflammatory biomarkers, the relationship between neutrophil counts and NLR and SHR were poorly explored in IS. We aimed to systemically and comprehensively explore the correlation between various blood inflammation markers (mainly neutrophil counts and NLR) and SHR.

**Methods:**

Data from 487 patients with acute IS(AIS) in Xiangya Hospital were retrospectively reviewed. High/low SHR groups according to the median of SHR (≤1.02 versus >1.02). Binary logistic regression analysis was used to evaluate the correlation between neutrophil counts and NLR and high SHR group. Subgroup analyses were performed in the TOAST classification and functional prognosis.

**Results:**

The neutrophil counts and NLR were all clearly associated with SHR levels in different logistic analysis models. In the subgroup analysis of TOAST classification, the higher neutrophil counts and NLR were the independent risk factors for high SHR patients with large-artery atherosclerosis (LAA) (neutrophil: adjusted OR:2.047, 95% CI: 1.355-3.093, P=0.001; NLR: adjusted OR:1.315, 95% CI: 1.129-1.530, P<0.001). The higher neutrophil counts were the independent risk factor for high SHR patients with cardioembolism (CE) (adjusted OR:2.413, 95% CI: 1.081-5.383, P=0.031). ROC analysis showed that neutrophil counts was helpful for differentiating high SHR group with CE and low SHR group with CE (neutrophil: AUC =0.776, P=0.002). However, there were no difference in levels of neutrophil counts and NLR between patients with SVO and without SVO. The higher neutrophil counts and NLR independently associated with high SHR patients with mRS ≤2 at 90 days from symptom onset, (neutrophil: adjusted OR:2.284, 95% CI: 1.525-3.420, P<0.001; NLR: adjusted OR:1.377, 95% CI: 1.164-1.629, P<0.001), but not in patients with mRS >2.

**Conclusions:**

This study found that the neutrophil counts and NLR are positively associated with SHR levels in AIS patients. In addition, the correlation between neutrophil counts and NLR and different SHR levels are diverse according to TOAST classification and functional prognosis.

## 1 Introduction

In recent decades, stroke has been the first leading cause of mortality and disability in China and imposes a substantial burden on family, society, and economy (1). Ischemic stroke (IS) accounts for ~81.9% of hospitalizations in all strokes in China (2). With the acceleration of China’s life expectancy and aging process, incidence of IS shows an increasing trend. Accordingly, how to improve prevention and treatment of IS has been a great concern.

Numerous studies have demonstrated that the immunity and inflammation play key roles in stroke (3, 4). Not only immune cells, but also cytokines and biochemical blood markers are involved in the mechanisms of IS progression. However, in daily practice, the assays of cytokines and immune cells are expensive and not widely available in hospitals. In turn, the assays of whole blood counts, such as white blood cells (WBC), neutrophil counts, and lymphocyte counts, have the advantages of speed, simplicity, and lower cost. In addition, whole blood counts as systemic inflammatory markers, which can provide valuable assessment for inflammatory response. Current studies have shown the important significance of whole blood counts, such as WBC, neutrophil counts, lymphocyte counts, and their combination ratios (neutrophil-lymphocyte ratio [NLR]), as markers of inflammation in AIS (5–7). Moreover, recent evidence also showed higher NLR was a predictor of stroke-associated pneumonia (8).

To our knowledge, almost half of IS patients may have stress hyperglycemia (9). Previous study found that acute hyperglycemia is an independent risk factor for in-hospital mortality and poor functional outcome after IS (10), regardless of the type of stroke treatment (11, 12), but with significance difference among diabetic and non-diabetic patients (13). In addition, hyperglycemia promoted the release of proinflammatory factors such as tumor necrosis factor-α(TNF-α) and interleukin-6 (IL-6) *in vitro* (14). A number of studies used the stress hyperglycemia ratio (SHR) as a tool to evaluate stress hyperglycemia (15, 16). Moreover, many studies have been found that SHR is associated with poor prognosis in AIS patients (17–19). Recent evidence revealed that elevated SHR was a clinical predictor of stroke-associated pneumonia (9).

However, up to now, the relationship between SHR and blood routine inflammatory indicators were poorly explored in AIS. This research aimed to systemically and comprehensively explore the correlation between various blood inflammation markers (mainly neutrophil counts and NLR) and SHR.

## 2 Materials and methods

### 2.1 Study participants

This study included consecutive AIS patients seen between July 2020 and September 2022 in Changsha Xiangya Hospital. AIS was defined by diffusion-weighted imaging (DWI) images. Inclusion criteria: (1) age ≥18 years old, (2) disease onset ≤ 14 days. Exclusion criteria: (1) Patients who no fasting blood glucose (FBG) and glycated hemoglobin (HbA1c), (2) Patients who no WBC, neutrophil counts and lymphocyte counts at admission, (3) Patients with other neurological diseases, (4) liver or renal failure.

### 2.2 Clinical assessments

We collected demographic variables (including age and sex), and vascular risk factors, including systolic blood pressure (SBP), diastolic blood pressure (DBP), history of stroke, history of hypertension, history of diabetes mellitus (DM), history of coronary heart disease (CAD), history of Hyperlipidemia, history of smoking and drinking. The definition of vascular risk factors was the same as the previous studies (20). Laboratorial findings included WBC, neutrophil, lymphocyte, triglycerides (TG), total cholesterol (TC), low-density lipoprotein (LDL), high-density lipoprotein (HDL), FBG, HbA1c, and homocysteine (Hcy).

Stroke severity at admission was assessed with the National Institutes of Health Stroke Scale (NIHSS) score, mild stroke: NIHSS score <6, moderate to severe stroke: NIHSS score of ≥6 (21). Discharge functional outcome was assessed with a modified Rankin Scale (mRS) score, good functional outcome: mRS score ≤ 2, poor functional outcome: mRS score of >2 (22). Etiology of ischemic stroke was assessed with Trial of Org 10172 in Acute Stroke Treatment (TOAST) (23).

### 2.3 Definition of stress hyperglycemia ratio

Fasting venous blood samples were collected within 24 hours after admission, and SHR was calculated from the following formula: FBG (mmol/L)/HbA1c (%) (24).

### 2.4 Statistical methods

SPSS 26.0, GraphPad Prism 8 and R software version R 4.2.1 were used to analyze statistical data and plot of the data. Categorical and continuous data were showed as counts and percentage (%) and medians [interquartile range (IQR), respectively. The Mann-Whitney U test was used to analyze continuous variables, and chi-squared test was used to analyze for categorical variables.

high/low SHR groups according to the median of SHR (≤1.02 versus >1.02). Binary logistic regression models were used to evaluate the differences among WBC, neutrophil counts, lymphocyte counts and NLR with different SHR groups. Three models were used by logistic regression. Spearman rank correlation test was used for correlation analyses among WBC, neutrophil counts, lymphocyte counts, NLR, and SHR. We performed subgroup analysis according to function prognosis at discharge and TOAST classification. Receiver operating characteristic (ROC) curve analysis was used to evaluate the values of neutrophil counts for differentiating high SHR group with CE and low SHR group with CE. Statistical significance was set at a 2-tailed P value <0.05.

## 3 Results

### 3.1 Baseline characteristics

A total of 487 AIS patients (male=320(65.7%); female=167(34.3%); median age=61 years) were enrolled in this study. The median of SHR was 1.02. High SHR group was associated with high frequency of DM, Hyperlipidemia, and higher SBP and DBP values. Simultaneously, High SHR group had higher levels of blood WBC, neutrophil counts, NLR, TG, TC, HDL, LDL, FBG, HbA1c, and lower levels of lymphocyte counts and Hcy levels. High SHR group had higher mRS score (**Table 1**).

**Table 1:** Baseline characteristics of patients with ischemic stroke according to different SHR levels.

Variables	Total	SHR		P-value
		Low SHR group (n=243) (≤1.02)	High SHR group(n=244) (>1.02)	
Age, years	61(53-69)	61 (53-70)	60 (54-69)	0.825
Sex (male, N, %)	320(65.7%)	163 (67.1%)	157 (64.3%)	0.525
Stroke, (N, %)	81(16.6%)	37 (15.2%)	44 (18.0%)	0.406
HBP, (N, %)	375(77.7%)	181 (74.5%)	194 (79.5%)	0.188
DM, (N, %)	168(34.5%)	63 (25.9%)	105(43.0%)	P<0.001
Hyperlipidemia, (N, %)	184(37.8%)	76 (31.3%)	108 (44.3%)	0.003
CAD, (N, %)	90(18.5%)	44 (18.1%)	46(18.9%)	0.832
Smoking, (N, %)	235(48.5%)	120 (49.4%)	116 (47.5%)	0.684
Drinking, (N, %)	167(34.3%)	81 (33.3%)	86 (35.21%)	0.657
SBP, mmHg	146.00(133.00-160.00)	142.00(130.00-154.75)	150.00 (137.75-166.00)	P<0.001
DBP, mmHg	85.00(76.00-94.00)	84.00(76.00-93.00)	86.00(77.00-95.25)	0.045
WBC, ×10^9^/L	7.00(5.80-8.40)	6.80 (5.70-8.00)	7.30 (5.80-9.10)	0.003
Platelet, ×10^9^/L	204.00(164.00-244.00)	203.50 (164.25-242.75)	204.00(126.75-246.00)	0.909
Neutrophil, ×10^9^/L	4.50(3.60-5.90)	4.25 (3.50-5.20)	5.10(3.70-6.70)	P<0.001
lymphocyte, ×10^9^/L	1.60(1.20-2.00)	1.60(1.30-2.10)	1.50(1.10-1.90)	0.001
NLR	2.93(2.00-4.41)	2.57(1.92-3.59)	3.32(2.14-5.19)	P<0.001
TG, mmol/L	1.60(1.15-2.24)	1.47 (1.11-2.05)	1.75(1.24-2.56)	P<0.001
TC, mmol/L	4.51(3.66-5.39)	4.24(3.55-5.07)	4.69 (3.82-5.60)	0.003
HDL, mmol/L	1.04(0.87-1.23)	1.00 (0.84-1.17)	1.07 (0.90-1.28)	0.001
LDL, mmol/L	2.82(2.25-3.42)	2.63(2.16-3.25)	2.96(2371-3.46)	0.001
FBG, mg/dl	6.22(5.18-8.28)	5.20(4.75-5.78)	7.86(6.50-11.39)	P<0.001
Homocysteine, µmol/L	13.57(11.12-16.74)	13.87 (11.56-16.90)	12.99 (10.98-16.60)	0.047
HbA1c (%), median (IQR)	5.90(5.50-7.20)	5.90(5.50-6.40)	6.10 (5.60-7.95)	0.019
SHR,median (IQR)	1.02(0.88-1.26)	0.88(0.82-0.94)	1.26(1.10-1.47)	P<0.001
NIHSS at admission, median (IQR)	4(2-7)	4(2-7)	4(2-7)	0.115
mRS at discharge, median (IQR)	2(1-3)	2(2-3)	2(2-4)	0.045
TOAST Etiology				0.565
LAA	287(58.9%)	137(56.3%)	150(61.4%)	
CE	46(9.4%)	24(9.8%)	22(9.0%)	
SVO	109(22.4%)	56(23.0%)	53(21.7%)	
SOE	15(3.1%)	4(2.8%)	8(3.2%)	
SUE	30(6.2%)	19(7.8%)	11(4.5%)	

SHR, FBG (mmol/L)/HbA1c (%); HBP, hypertension; DM, diabetes mellitus; CAD, coronary heart disease; WBC, white blood cells; NLR, neutrophil-to-lymphocyte ratio; SBP, systolic blood pressure; DBP, diastolic blood pressure; TC, total cholesterol; TG, triglycerides; HDL, high density lipoprotein; LDL, low density lipoprotein. FBG, fasting blood glucose; HbA1c, glycated hemoglobin; IQR, interquartile range; NIHSS, Initial stroke severity was assessed with the National Institutes of Health Stroke Scale; mRS, modified Rankin Scale; TOAST, Trial of Org 10172 in Acute Stroke Treatment; LAA, large-artery atherosclerosis; CE, cardioembolism; SVO, small-vessel occlusion; SOE, other determined etiology; SUE undetermined etiology.

### 3.2 Differences of WBC, neutrophil counts, lymphocyte counts, and NLR between low SHR group and high SHR group

**Figure 1:**
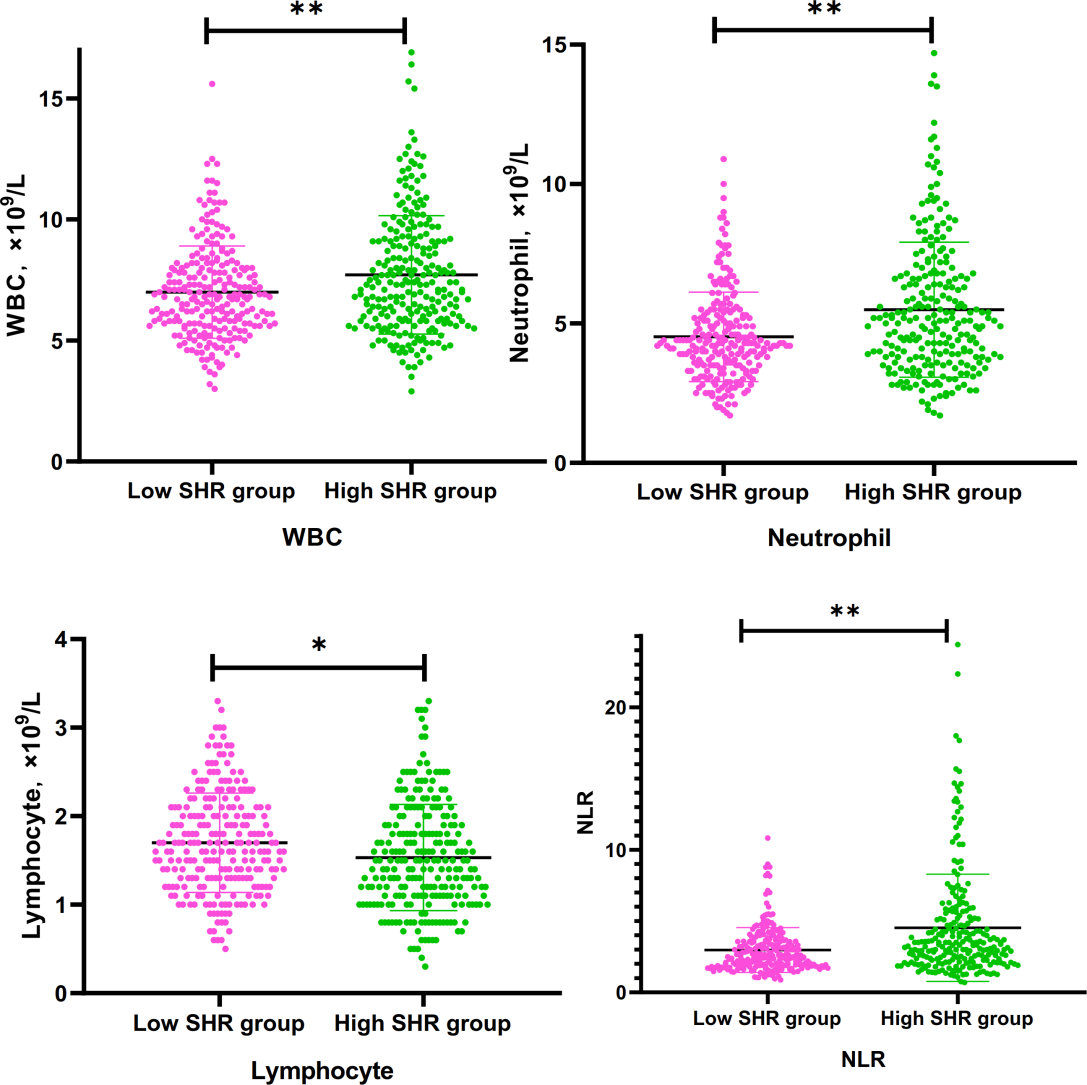
Differences of WBC, Neutrophil. lymphocyte, and NLR between low SHR group and hig SHR group. SHR-fasting blood glucose (mmol/L)/HbA1c (%); WBC, white blood cells; NLR, neutrophil-to-lymphocyte ratio. *P<095; **p<0.001.

There were 243 AIS patients in low SHR group, and 244 AIS patients in high SHR group. WBC, neutrophil counts, lymphocyte counts, and NLR as continuous variables, all the four parameters were independently associated with high SHR group in different logistic analysis models. When the first quartile was regarded as reference, the fourth quartiles of WBC, neutrophil counts, lymphocyte counts, and NLR were significantly associated with high SHR group in different logistic analysis models. In addition, the second and third quartiles of lymphocyte levels were independently associated with high SHR group in different logistic analysis. The third quartiles of NLR independently associated with high SHR group in model1 (**Table 2** and **Figure 1**).

**Table 2:** Unadjusted and adjusted analyses of WBC, neutrophil counts, lymphocyte counts, and NLR with high SHR group.

Variables	Cell counts(10^9^/L) in quartiles	Unadjusted analysis		Model 1		Model 2	
		OR (95% CI)	p value	OR (95% CI)	p value	OR (95% CI)	p value
WBC, ×10^9^/L	<5.80	Reference	0.006		0.005		0.022
	5.80-7.00	1.041(0.627-1.727)	0.877	1.067(0.640-1.780)	0.804	1.117(0.642-1.945)	0.695
	7.00-8.40	1.051(0.630-1.753)	0.850	1.084(0.644-1.826)	0.761	1.045(0.595-1.834)	0.879
	≥8.40	2.193(1.315-3.657)	0.003	2.266(1.342-3.825)	0.002	2.130(1.217-3.729)	0.008
Neutrophil, ×10^9^/L	<3.60	Reference	P<0.001		P<0.001		P<0.001
	3.60-4.50	0.763(0.455-1.279)	0.305	0.787(0.466-1.330)	0.371	0.705(0.400-1.241)	0.226
	4.50-5.90	1.376(0.828-2.285)	0.218	1.413(0.845-2.362)	0.188	1.202(0.687-2.104)	0.519
	≥5.90	2.640(1.569-4.441)	P<0.001	2.708(1.598-4.590)	P<0.001	2.451(1.392-4.315)	0.002
Lymphocyte, ×10^9^/L	<1.20	Reference	0.009		0.005		P<0.001
	1.20-1.60	0.593(0.354-0.992)	0.047	0.581(0.346-0.975)	0.04	0.434(0.245-0.767)	0.004
	1.60-2.00	0.593(0.354-0.992)	0.007	0.445(0.258-0.769)	0.004	0.355(0.197-0.639)	0.001
	≥2.00	0.439(0.264-0.732)	0.002	0.404(0.238-0.685)	0.001	0.295(0.164-0.530)	P<0.001
NLR	<2.00	Reference	P<0.001		P<0.001		P<0.001
	2.00-2.93	1.510(0.903-2.524)	0.166	1.609(0.955-2.713)	0.074	1.591(0.904-2.800)	0.107
	2.93-4.21	1.633(0.975-2.738)	0.063	1.710(1.015-2.880)	0.044	1.634(0.921-2.898)	0.093
	≥4.21	3.856(2.256-6.590)	P<0.001	4.160(2.405-7.196)	P<0.001	4.592(2.533-8.322)	P<0.001
WBC, ×10^9^/L		1.163(1.069-1.267)	P<0.001	1.170(1.073-1.276)	P<0.001	1.165(1.063-1.277)	0.001
Neutrophil, ×10^9^/L		1.274(1.156-1.404)	P<0.001	1.280(1.16-1.412)	P<0.001	1.280(1.155-1.419)	P<0.001
Lymphocyte, ×10^9^/L		0.608(0.445-0.831)	0.002	0.576(0.414-0.769)	0.001	0.475(0.329-0.684)	P<0.001
NLR		1.279(1.165-1.403)	P<0.001	1.289(1.172-1.417)	P<0.001	1.321(1.195-1.460)	P<0.001

Model 1 adjusted for age, sex. Model2 adjusted for age, sex, diabetes mellitus, Hyperlipidemia, SBP, DBP, TG, TC, HDL, LDL, and Homocysteine. OR, odds ratio; WBC, white blood cells; NLR, neutrophil-to-lymphocyte ratio; SBP, systolic blood pressure; DBP, diastolic blood pressure; TC, total cholesterol; TG, triglycerides; HDL, high density lipoprotein; LDL, low density lipoprotein. TOAST, Trial of Org 10172 in Acute Stroke Treatment; LAA, large-artery atherosclerosis; CE, cardioembolism; SVO, small-vessel occlusion.

To assess the linear association among the WBC, neutrophil counts, lymphocyte counts, and NLR with SHR, we constructed Spearman rank correlation analysis (**Supplementary figure 1**). Linear positive correlations among the WBC (r=0.18, p<0.001), neutrophil counts (r=0.230, p<0.001), and NLR (r=0.210, p<0.001) with SHR, respectively. The correlation between lymphocyte counts and SHR was not statistically significant (r=-0.087, p=0.056). The result suggested that the strongest association was observed between neutrophil counts and SHR.

### 3.3 Subgroup analyses were conducted between neutrophil counts and NLR with different SHR levels

Subgroup analyses were performed according to TOAST classification (LAA vs. SVO vs. CE) of ischemic stroke etiology and function prognosis at discharge and 90 days from stroke outset (mRS≤ 2 vs. mRS >2).

**Figure 2:**
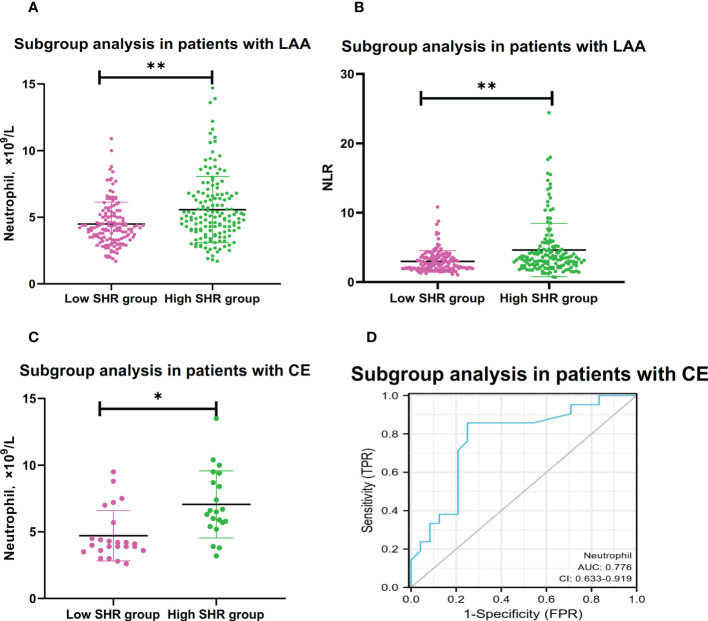
Subgroup analyses for association between neutrophil and NLR with high SHR group.**(A)** Comparison of neutrophil counts between low SHR group and high SHR group in patients with LAA. **(B)** Comparison of NLR between low SHR group and high SHR group in patients with LAA. **(C)** Comparison of neutrophil counts between low SHR group and high SHR group in patients with CE. **(D)** ROC analysis of predication performance of neutrophil for high SHR group in patients with CE. (Neutrophil: AUC 0.776, 95% CI 0.633-0.919; P=0.002, specificity 0.750, sensitivity 0.857; optimal cut-off: 4.850) (*P <0.05, **P <0.001). NLR, neutrophil-to-lymphocyter ratio: SHR-fasting blood glucose AUC: Areas under the receiver operating characteristics curve (ROC) curves.

In the subgroup analysis of TOAST classification, high SHR patients with large-artery atherosclerosis (LAA) had clearly higher levels of neutrophil counts and NLR than low SHR patients with LAA; Multivariable logistic regression analysis showed the higher levels of neutrophil counts and NLR were the independent risk factors for high SHR patients with LAA (neutrophil:adjusted OR:2.047, 95% CI: 1.355-3.093, P=0.001; NLR: adjusted OR:1.315, 95% CI: 1.129-1.530, P<0.001) (**Table 3** and **Figures 2A, B**). Multivariable logistic regression analysis also showed high SHR patients with cardioembolism (CE) had clearly higher neutrophil counts than low SHR patients with CE (adjusted OR:2.413, 95% CI: 1.081-5.383, P=0.031) (**Table 3** and **Figure 2C**). However, there’s no difference in neutrophil counts and NLR between high SHR patients with small-vessel occlusion (SVO) than low SHR patients with SVO (**Table 3**). ROC analysis showed that neutrophil counts was helpful for differentiating high SHR group with CE and low SHR group with CE (neutrophil: AUC =0.776, 95%CI 0.633-0.919; P=0.002, specificity 0.750, sensitivity 0.857; optimal cut-off: 4.850, **Figure 2D**).

**Table 3:** Unadjusted and adjusted analyses of neutrophil counts and NLR with high SHR group according to TOAST classification of ischemic stroke etiology.

Variables	Unadjusted model		Multivariable- Model	
	OR (95% CI)	p value	OR (95% CI)	p value
Neutrophil, ×10^9^/L				
LAA	1.296(1.141-1.472)	P<0.001	2.047(1.355-3.093)	0.001
CE	1.466(1.093-1.965)	0.011	2.413(1.081-5.383)	0.031
SVO	0.974(0.744-1.274)	0.847	0.974(0.744-1.274)	0.847
NLR				
LAA	1.298(1.143-1.473)	P<0.001	1.315(1.129-1.530)	P<0.001
CE	1.332(1.050-1.690)	0.018	1.436(0.938-2.197)	0.095
SVO	1.005(0.805-1.255)	0.966	1.035(0.818-1.308)	0.777

LAA Multivariable- Model: adjusted for:sex, age, hyperlipidemia, DM, SBP, WBC, TG, TC, HDL, LDL.CE Multivariable- Model: adjusted for sex, age, DM, SBP, WBC, TC.SVO Multivariable- Model: adjusted for:sex, age, DM. TOAST: Trial of Org 10172 in Acute Stroke Treatment; LAA, large-artery atherosclerosis; CE, cardioembolism; SVO, small-vessel occlusion; OR, odds ratio; NLR, neutrophil-to-lymphocyte ratio; DM, diabetes mellitus; SBP, systolic blood pressure; WBC, white blood cells; TG, triglycerides; TC, total cholesterol; HDL, high density lipoprotein; LDL, low density lipoprotein.

In the subgroup analysis of discharge function prognosis, there’s no difference in the subgroup analysis of function prognosis. Independently of mRS status, high SHR correlates with higher neutrophil counts and higher NLR (**Table 4**).

**Table 4:** Unadjusted and adjusted analyses of neutrophil and NLR with high SHR group according to discharge functional outcomes subgroup.

Variables	Unadjusted model		Multivariable- Model	
	OR (95% CI)	p value	OR (95% CI)	p value
Neutrophil, ×10^9^/L				
mRS≤2	1.172(1.035-1.326)	0.012	1.176(1.029-1.344)	0.018
mRS>2	1.413(1.196-1.670)	P<0.001	2.096(1.308-3.358)	0.002
NLR				
mRS≤2	1.218(1.07-1.385)	0.003	1.220(1.069-1.323)	0.003
mRS>2	1.319(1.148-1.515)	P<0.001	1.344(1.132-1.596)	0.001

mRS≤2 Multivariable-Model: adjusted for: age, sex, DM, Hyperlipidemia, SBP, DBP, TC, LDL; mRS>2 Multivariable- Model: adjusted for: sex, age, DM, SBP, WBC, HDL. mRS: modified Rankin Scale; NLR, neutrophil-to-lymphocyte ratio; OR, odds ratio; DM, diabetes mellitus; SBP, systolic blood pressure; WBC, white blood cells; TC, total cholesterol; HDL, high density lipoprotein; LDL, low density lipoprotein.

In the subgroup analysis of 90 days function prognosis, the correlation of the high SHR correlates with higher neutrophil counts and higher NLR were dependent on different prognosis. Multivariable logistic regression analysis showed the higher levels of neutrophil counts and NLR as the independent risk factors for High SHR patients with mRS ≤2, (neutrophil: adjusted OR:2.284, 95% CI: 1.525-3.420, P<0.001; NLR: adjusted OR:1.377, 95% CI: 1.164-1.629, P<0.001), but not in high SHR patients with mRS >2. (**Table 5**).

**Table 5:** Unadjusted and adjusted analyses of neutrophil and NLR with high SHR group according to 90 days functional outcomes subgroup.

Variables	Unadjusted model		Multivariable- Model	
	OR (95% CI)	p value	OR (95% CI)	p value
Neutrophil, ×10^9^/L				
mRS≤2	1.313(1.156-1.492)	P<0.001.	2.284(1.525-3.420)	P<0.001
mRS>2	1.397(1.097-1.1778)	0.007	1.867 (0.900-3.873)	0.093
NLR				
mRS≤2	1.367(1.181-1.583)	P<0.001	1.377(1.164-1.629)	P<0.001
mRS>2	1.246(1.032-1.504))	0.022	1.204(0.935-1.552)	0.150

mRS≤2 Multivariable-Model: adjusted for: age, sex, DM, Hyperlipidemia, SBP, WBC, TG, TC, HDL, LDL; mRS>2 Multivariable-Model: adjusted for: age, sex, DM, SBP, DBP, WBC, LDLC. mRS: modified Rankin Scale; NLR, neutrophil-to-lymphocyte ratio; OR, odds ratio; DM, diabetes mellitus; SBP, systolic blood pressure; WBC, white blood cells; TG, triglycerides; TC, total cholesterol; HDL, high density lipoprotein; LDL, low density lipoprotein.

## 4 Discussion

The current study is the first study that systemically and comprehensively investigated the correlation between various systemic blood inflammatory factors and stress hyperglycemia. First, our study found that all four parameters of WBC, neutrophil counts, lymphocyte counts, and NLR were independently associated with high SHR group; second, among different stroke etiology patients, in accordance with the TOAST classification, there’s not always a correlation between SHR levels, neutrophil counts and NLR, and neutrophil counts was helpful for differentiating high SHR group with CE and low SHR group with CE; last, the correlation of the high SHR correlates with higher neutrophil counts and higher NLR were dependent on different functional prognosis.

In patients with IS, elevated neutrophil counts and NLR predicted poor outcome and stroke recurrence (25). Consistent with previous studies, higher neutrophil counts and NLR were predictors for worse functional outcome in AIS patients in our study (**Supplementary Figure 2**). Acute cerebral ischemia triggers the rapid inflammatory reaction. After IS, the integrity of the blood-brain barrier (BBB) is disrupted. Destruction of the BBB promotes the migration of peripheral immune cells to the brain. Neutrophils are rapidly recruited into cerebral tissue. The study found neutrophil recruitment in leptomeninges from 6 h in an animal model of IS, in the cortical basal lamina from 15 h, and in the cerebral parenchyma at 24 h by confocal microscopy in mice and human IS (26). Neutrophil extracellular traps (NETs) were released by neutrophils, which can promote thrombus formation, exacerbate injury of neurons, foster inflammation, and impair vascular remodeling after IS (27–29). Low lymphocyte counts were demonstrated to have a neuroprotective effect in AIS (30, 31). NLR is defined by neutrophil counts divided by lymphocyte counts. Previous studies showed that NLR could predict the clinical prognosis, hemorrhagic transformation (HT), and stroke-associated pneumonia in IS patients (8, 32, 33).

The catecholamines, inflammatory cytokines, and IR act synergistically to promote stress hyperglycemia in different diseases (34, 35). SHR, defined as FBG/HbA1c ratio, was used to represent the state of stress hyperglycemia. SHR was associated with functional outcome, complications, HT, and stroke recurrence in AIS patients (9, 18, 36). Although the pathogenic mechanisms are not so clear, it was proposed that hyperactivated stress could trigger BBB breakdown, oxidative stress response, inflammation and cytokine release in stroke (19). Zhao et al. found that high glucose could promote inflammation of endothelial cell by hypoxia-inducible factor-1 alpha signaling pathway (37). Chronic hyperglycemia can promote oxidative stress and the chronic accumulation of advanced glycation end products (AGEs) (38). AGEs have been proved that could induce inflammatory activation in different diseases (39), such as AGEs increase interleukin (IL)-6 expression *via* NF-κB pathways (40). Although stress hyperglycemia has been shown to correlate with inflammatory cytokines, the assays of cytokines and immune cells are expensive and not widely available in hospitals. In turn, WBC, neutrophil counts, lymphocyte counts, and NLR are available and inexpensive biomarkers from routine laboratory data. Therefore, we explore the correlation between various blood inflammatory markers and different SHR levels. In our study, we found that high SHR levels (SHR>1.02) were clearly associated with higher levels of WBC, neutrophil counts, and NLR.

TOAST classification is the widest tool to determine IS etiology, which categorizes ischemic stroke into five etiological subtypes: LAA, CE, SVO, other determined etiology (SOE); and undetermined etiology (SUE), respectively (41). LAA, which is the most common subtype of IS, is primarily caused by atherosclerosis. Previous studies have found atherosclerosis is a chronic inflammatory disease, which can promote the formation, progression, and rupture of atherosclerotic plaque (42). Hyperglycemia promotes of formation AGEs, which could induce progression of atherosclerosis *via* inflammation and oxidative stress response (43). In our study, we found the higher levels of neutrophil counts and NLR were associated with high SHR levels in the IS patients with LAA. We speculated that stress hyperglycemia may promote the progression of atherosclerosis by activating peripheral blood lymphocytes and neutrophils and disrupting BBB in IS patients with LAA. Previous studies found that CE promoted more inflammatory cytokines release, is the most serious strokes, and has the worst prognosis compared to other IS etiologies (44, 45). Previous study indicated that inflammation might induce stress hyperglycemia by promoting hepatic gluconeogenesis (35). In our study, we found the higher levels of neutrophil counts were associated with high SHR levels in the IS patients with CE. We speculated that CE promoted more severe inflammation, and inflammation induced stress hyperglycemia. However, there were no difference in levels of neutrophil counts and NLR between patients with SVO and without SVO. The present study indicated that the association between neutrophil counts and NLR and stress hyperglycemia may be more likely to occur in IS patients with LAA and CE subtypes.

This study has several limitations. First, this study was performed in single time point, the association between dynamic changes of blood inflammatory factors and different SHR levels were expected in future. Second, the study was designed to collect clinical data only from Xiangya Hospital, which may result in the selection bias. Third, this study was observational, and the causal relationship cannot be clarified. Thus, prospective, multi-center studies were expected to clarify this relationship.

## 5 Conclusions

This study found that the high levels of neutrophil counts and NLR are positively associated with SHR levels in AIS patients. In addition, the correlation between neutrophil counts and NLR and different SHR levels are diverse according to TOAST classification of IS etiology and functional prognosis.

## Data availability statement

The original contributions presented in the study are included in the article/**Supplementary Material**. Further inquiries can be directed to the corresponding author.

## Ethics statement

The studies involving human participants were reviewed and approved by Xiangya Hospital Ethics Committee. The patients/participants provided their written informed consent to participate in this study.

## Author contributions

Concept and design: XF. Clinical data: XF, FY, MW, YL, TZ, ZL, QH, RT, JL, BZ, LC. Statistical analyses: XF and FY. Draft manuscript: XF and FY. JX reviewed the manuscript, and contributed to discussions. All authors contributed to the article and approved the submitted version.
