# Supplementary material for: The association between neutrophil counts and neutrophil-to-lymphocyte ratio and stress hyperglycemia in patients with acute ischemic stroke according to stroke etiology
Source: Front Endocrinol (Lausanne). 2023 Mar 16;14:1117408. doi: 10.3389/fendo.2023.1117408 (PMC10060840; doi:10.3389/fendo.2023.1117408)
Supplement: Supplementary file 1 [file Image_1.pdf]

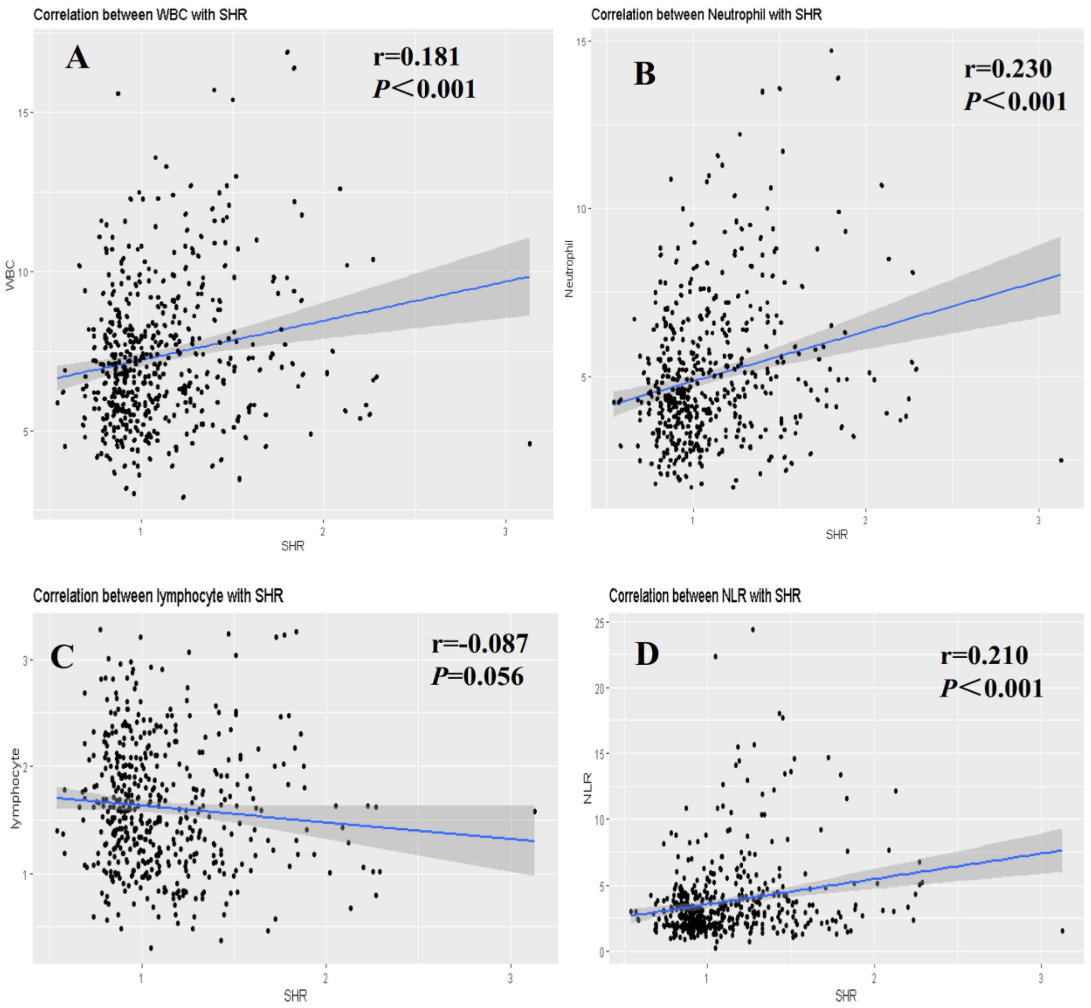

Supplementary Figure1. correlation WBC(A), Neutrophil(B), Lymphocyte(C) and NLR(D) with SHR in AIS. SHR=fasting blood glucose (mmol/L)/HbA1c (%); WBC: white blood cells; NLR, neutrophil-to-lymphocyte ratio; AIS: acute ischemic stroke.

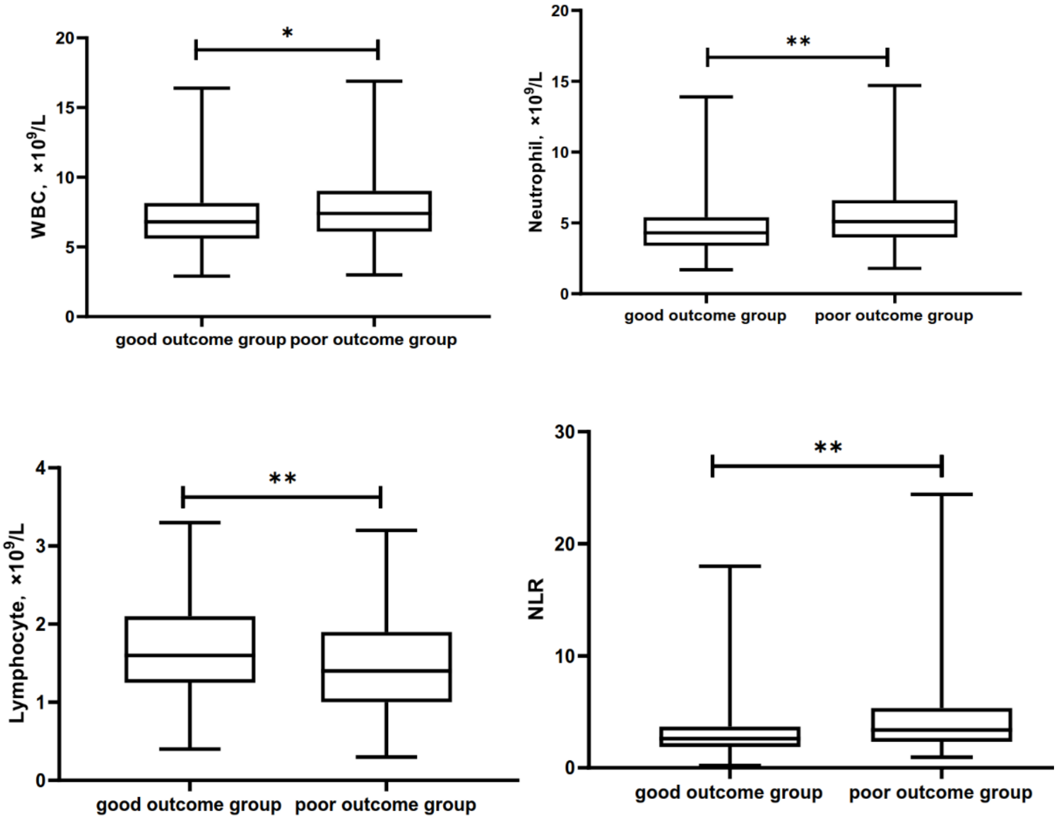

Supplementary Figure 2: The Differences of WBC, Neutrophil, lymphocyte, and NLR between good outcome group and poor outcome group at discharge. WBC: white blood cells; NLR, neutrophil-to-lymphocyte ratio; mRS: modified Rankin Scale. *P<0.05;p<0.001; good outcome group(mRS≤2); poor outcome group(mRS>2).**
